# Whole Transcriptomic Analysis Provides Insights into Molecular Mechanisms for Toxin Biosynthesis in a Toxic Dinoflagellate *Alexandrium catenella* (ACHK-T)

**DOI:** 10.3390/toxins9070213

**Published:** 2017-07-05

**Authors:** Yong Zhang, Shu-Fei Zhang, Lin Lin, Da-Zhi Wang

**Affiliations:** State Key Laboratory of Marine Environmental Science/College of the Environment and Ecology, Xiamen University, Xiamen 361102, China; foryzhy@gmail.com (Y.Z.); shufeizhang@qq.com (S.-F.Z.); linlin1982@xmu.edu.cn (L.L.)

**Keywords:** dinoflagellate, *Alexandrium catenella*, paralytic shellfish toxins, toxin biosynthesis, cell cycle, RNA-seq

## Abstract

Paralytic shellfish toxins (PSTs), a group of neurotoxic alkaloids, are the most potent biotoxins for aquatic ecosystems and human health. Marine dinoflagellates and freshwater cyanobacteria are two producers of PSTs. The biosynthesis mechanism of PSTs has been well elucidated in cyanobacteria; however, it remains ambiguous in dinoflagellates. Here, we compared the transcriptome profiles of a toxin-producing dinoflagellate *Alexandrium catenella* (ACHK-T) at different toxin biosynthesis stages within the cell cycle using RNA-seq. The intracellular toxin content increased gradually in the middle G1 phase and rapidly in the late G1 phase, and then remained relatively stable in other phases. Samples from four toxin biosynthesis stages were selected for sequencing, and finally yielded 110,370 unigenes, of which 66,141 were successfully annotated in the known databases. An analysis of differentially expressed genes revealed that 2866 genes altered significantly and 297 were co-expressed throughout the four stages. These genes participated mainly in protein metabolism, carbohydrate metabolism, and the oxidation-reduction process. A total of 138 homologues of toxin genes were identified, but they altered insignificantly among different stages, indicating that toxin biosynthesis might be regulated translationally or post-translationally. Our results will serve as an important transcriptomic resource to characterize key molecular processes underlying dinoflagellate toxin biosynthesis.

## 1. Introduction

Dinoflagellates are not only important primary producers in marine ecosystems, but also major causative agents producing paralytic shellfish toxins (PSTs) [1]. Comprising saxitoxin (STX) and its recorded 57 analogs, PSTs act as the most potent and selective voltage-gated sodium channel blockers [2,3]. They can cause human neural system syndromes, occasionally even death, and lead to approximately 2000 toxicosis cases annually worldwide [4]. Owing to their severe threat to human health and serious damage to the marine ecosystem, STX and its biosynthesis mechanism have aroused wide public concern.

Recently, the gene cluster *sxt* for STX synthesis has been elucidated in cyanobacteria, another well-known organism producing STX, followed by the identification of *sxt* clusters in several other cyanobacterial species [5,6,7,8]. With a length ranging from 25.7 kb to 36 kb, the clusters encode 24 to 32 proteins that participate in toxin biosynthesis [9]. Integrating their functions and the intermediates produced by each reaction, the biosynthesis pathway is proposed and refined in cyanobacteria [5,7,9]. However, only two genes—*sxtA* and *sxtG*—which catalyze the first two reactions of toxin biosynthesis, have been clarified in dinoflagellates [10,11]. Two sorts of dinoflagellate *sxtA* isoforms are identified, including a long transcript containing *sxtA1*-*A4* catalytic domains and a short transcript containing only *sxtA1*-*A3* domains [10]. The domain *sxtA4* is speculated to be essential for toxin biosynthesis, and this domain together with *sxtG* is restricted to dozens of toxin-producing dinoflagellate species [12,13,14]. As for other *sxt* genes, although some homologues of cyanobacterial genes are acquired in several studies, little is known about their exact functions in toxin biosynthesis [10,14,15].

Like many other eukaryotes, dinoflagellates also possess a typical eukaryotic cell cycle of G1-S-G2/M [16]. Previous studies have demonstrated that the biosynthesis of STX may be in connection with and regulated by the cell cycle. In *Alexandrium fundyense*, toxin production proceeds mainly during the early G1 phase, and the toxin content remains relatively constant in other cell cycle phases [17,18]. In *Alexandrium catenella*, rising and falling toxin content is observed within a cell cycle, and the highest content is found in the S phase, indicating the simultaneous biosynthesis of toxin and DNA in the cells [19]. The synthesis of toxin is discontinuous in another *A. catenella* CAWD44 cell cycle, with the highest toxin content and synthesis rate in the G2/M phase [20]. This discovery is inconsistent with the previously universal understanding that toxin biosynthesis occurs only in the daytime [21,22]. Strain and species specific differences are proposed to explain this distinction, which leads to a more complex toxin biosynthesis than previously thought [20]. Recently, toxin production has been demonstrated to occur in the S phase of *Alexandrium tamarense* based on the treatment result of metabolic inhibitors on the cell cycle and toxin content [23,24]. Based on these findings, some attempts were made to seek toxin biosynthesis-related genes based on the potential relevancies between cell cycle and toxin content. Using differential display technology, genes with altered expressions in the toxin-producing G1 phase were screened; however, none of these genes were found to be directly related to toxin biosynthesis [25]. Three genes, *Sam*, *Sahh*, and *Map*, are proposed to participate in toxin production in the G2/M phase, but their exact functions are elusive [20]. By comparing protein profiles at different toxin biosynthesis stages, nine proteins are identified to potentially participate in toxin biosynthesis; however, their encoding genes are not confirmed [26].

Our previous study on the transcriptomic analysis of a toxigenic *A. catenella* (ACHK-T) and its non-toxic mutant (ACHK-NT) identifies a dozen toxin-related genes. Nonetheless, only the *sxtA* gene presents different expression between the two strains [14]. Thus, a further investigation is required to unveil the toxin-related genes and the mechanism of STX biosynthesis in dinoflagellates. Considering the correlation between cell cycle and toxin biosynthesis, we therefore carried out a transcriptome comparison of ACHK-T at different toxin biosynthesis stages within the cell cycle, and discussed the regulation mechanism of toxin biosynthesis in marine dinoflagellates.

## 2. Results

### 2.1. Cell Cycle and Intracellular Toxin Content

**Figure 1:**
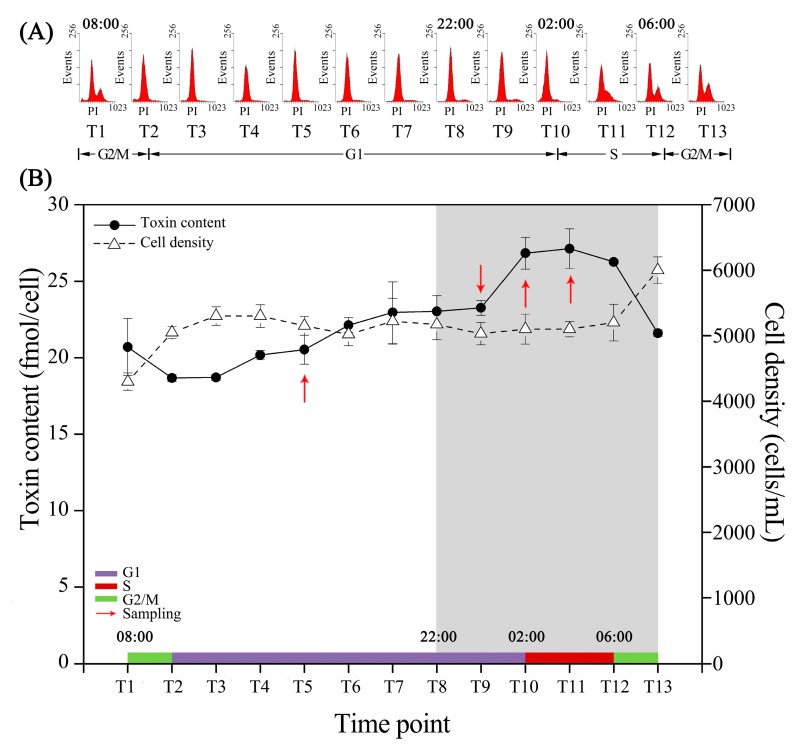
Cell cycle phase, cell density and intracellular toxin content of ACHK-T. The time interval between each time point is 2 h. (**A**) Cell cycle phase distribution. The X-axis is the relative amount of DNA and Y-axis is the number of cells in a sample containing a particular amount of DNA. The peak patterns indicate different cell cycle phases. (**B**) Curves of cell density and intracellular toxin content. The colorized line above the X-axis shows the cell cycle phase distribution obtained in subfigure (A). The grey box corresponds to the dark period and the red arrows represent the time points when samples were collected for RNA-seq. The toxin contents were reported as the mean of biological triplicates with standard deviation. (*n* = 3, T5-vs-T9: *p*-value = 0.000; T9-vs-T10: *p*-value = 0.000; T10-vs-T11: *p*-value = 0.722; other *p*-values are shown in Supplementary Files S6).

Flow cytometry (FCM) analysis was carried out to analyze the cell cycle distribution. Cells of ACHK-T completed a whole cell cycle within 24 h (Figure 1A): the cells stayed in the G2/M phase when the light was turned on at T1 (08:00), followed by entering the G1 phase at T2 (10:00), and this phase lasted for 16 h until T10 (02:00) on the next day. Then, the S phase started, and proceeded until T12 (06:00), after which the cells entered the next G2/M phase.

Within the cell cycle, cell density maintained relatively constant, except for the T1 and T13 time points when the cell division occurred (Figure 1B). However, the results of the high-performance liquid chromatography (HPLC) analysis showed that the intracellular toxin content differed significantly (*p* < 0.05), and presented two increasing periods: a slow increase during the light period and a rapid enhancement 2 h after the dark period, which started at T8 (22:00). The latter period lasted for nearly 2 h, and the toxin content reached its maximum at T11 (04:00), after which the toxin maintained a stable concentration with slight variations before cell division began at T12 (06:00).

### 2.2. Transcriptome Sequencing and Assembly

The transcriptome analysis of ACHK-T using the Illumina Hiseq 2000 sequencing platform generated approximately 120 million reads for the four individual libraries, and 480,981,760 raw reads in total. After the removal of reads that contained unreadable nucleotides or adaptor sequences and reads with low quality, these libraries yielded 111,059,650 (T5), 108,902,332 (T9), 109,129,854 (T10), and 106,451,730 (T11) clean reads, with the Q20 percentage from each library ranging from 96.50 to 96.69. These clean reads were further assembled into 132,057 (T5), 131,742 (T9), 130,612 (T10), and 127,969 (T11) unigenes with average lengths of 751, 750, 736, and 751 bp. After pooling all of the reads from the four libraries, 111,871 unigenes were obtained in total, which possessed an average size of 993 bp and an N50 length of 1442 bp. The statistical details of the transcriptome sequences are given in Supplementary Table S1. The length of 35.60% of the unigenes ranged from 200 to 500 bp, and 54.16% varied from 500 to 2000 bp, while the length of about 10.24% was longer than 2000 bp. The size distribution of all of the unigenes is shown in Supplementary Figure S1.

### 2.3. Gene Functional Annotations

To annotate these genes, functional retrieving was carried out against the NCBI non-redundant protein sequences (NCBI NR), the Kyoto Encyclopedia of Genes and Genomes (KEGG), the Cluster of Orthologous Groups (COG), and the Swiss-Prot databases using BLASTX, and against the NCBI NT nucleotide database using BLASTN with a cut-off e-value of 1 × 10^−5^ (Supplementary File S5). The number of unigenes successfully annotated in at least one database was 74,261 (66.38% of all unigenes), and 72,936 unigenes obtained annotations in the NR database (Table 1). The distribution of significant homology matches in the NR database showed that 35.15% of the homologous sequences possessed e-values smaller than 10^−15^ (Supplementary Figure S2A), and 1124 (1.54%) unigenes presented sequence similarity higher than 80% (Supplementary Figure S2B). In respect of species specific distribution, 24.77% of transcripts had matches to gene sequences from *Aureococcus anophagefferens*, followed by *Guillardia theta* CCMP2712 (9.22%). The percentage of unigenes homologous to sequences from other species was 45.71% (Supplementary Figure S2C). It should be pointed out that only a small fraction of transcripts recovered in our study was mapped to dinoflagellate transcripts, owing to the poor annotations of current dinoflagellate expressed sequence tags (EST) or transcriptome data in the NCBI database, as described in a previous study [27].

To facilitate the overview of gene functions, all unigenes were subcategorized into the COG classification (Supplementary Figure S3), Gene Ontology (GO) categories (Supplementary Figure S4), and the KEGG Analysis (Supplementary Figures S5–S7).

### 2.4. Differentially Expressed Genes (DEGs) among Four Samples

**Figure 2:**
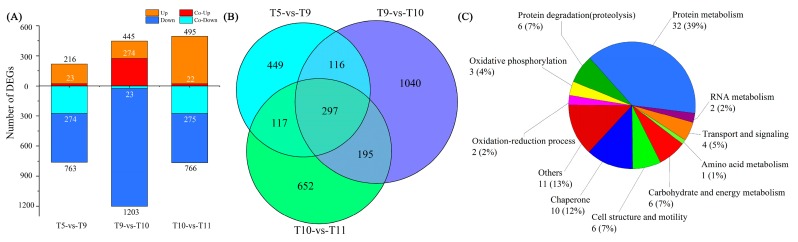
Differentially expressed genes (DEGs) among the datasets. (**A**) Histogram presentation of the number of upregulated and downregulated genes from each comparison. A total of 297 genes were co-expressed amongst the three comparisons. (**B**) Venn diagram showing the overlapping number of DEGs among the comparisons. (**C**) Functional classification of the co-expressed DEGs.

A criterion with a fold change ≥ 2 and false discovery rate (FDR) ≤ 0.001 was applied to judge the DEGs. Comparisons of the four samples were carried out sequentially in pairs, and 2866 DEGs in total were singled out. More genes were downregulated than upregulated in all of the comparisons (Figure 2A). Between each pair comparison, 449 genes were expressed exclusively in T5-vs-T9, 1,040 in T9-vs-T10, and 652 in T10-vs-T11 (Figure 2B). Of these DEGs, 297 genes were found co-expressed throughout three comparisons, and most of these genes were upregulated in T9-vs-T10, while they were downregulated in T5-vs-T9 and T10-vs-T11 (Figure 2A,B). These genes were principally associated with ‘protein metabolism’, ‘chaperone’, ‘carbohydrate and energy metabolism’, and ‘cell structure and motility’ (Figure 2C).

**Figure 3:**
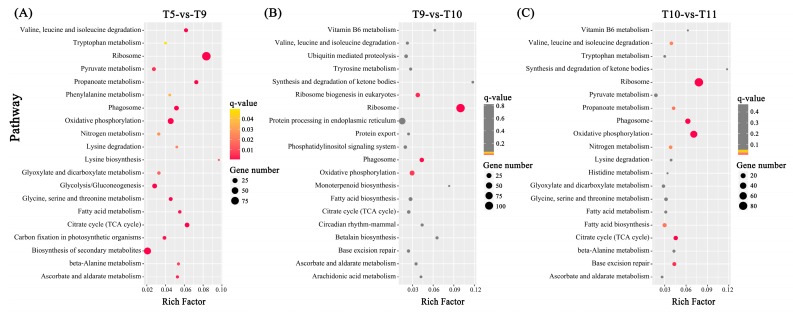
KEGG enrichment analysis of all DEGs from each comparison. The top 20 represented pathways are shown in the scatterplot for (**A**) comparison T5-vs-T9, (**B**) comparison T9-vs-T10 and (**C**) comparison T10-vs-T11. All enriched pathways are listed in Supplementary File S2. The rich factor is the ratio of DEGs number to the total gene number in a certain pathway. The size of the dots represent the gene number. The color of dots indicates the scopes of *q*-value (≤0.05) and the grey dots indicate the pathways were not significantly enriched (*q*-value > 0.05).

The GO classification of all of the DEGs into three main categories was performed (Supplementary Figure S8), and the detailed subcategorization for each comparison is shown in Supplementary File S1. Subsequently, a pathway enrichment analysis was carried out with the threshold *q*-value ≤ 0.05 by mapping all of the DEGs to the terms in the KEGG database and comparing with the whole transcriptome background (Supplementary File S2). The top 20 pathways of each comparison are shown in Figure 3. For T5-vs-T9, a total of 391 DEGs were assigned to 84 pathways, of which 20 pathways were significantly enriched. The most represented pathways encompassed ‘Ribosome’, ‘Oxidative phosphorylation’, and ‘Citrate cycle (TCA cycle)’. In T9-vs-T10, 563 DEGs involved in 75 pathways were obtained, with four significantly enriched pathways: ‘Ribosome’, ‘Phagosome’, ‘Ribosome biogenesis in eukaryotes’, and ‘Oxidative phosphorylation’. In T10-vs-T11, 72 KEGG pathways were related to 479 DEGs, and nine of these pathways were observably enriched. ‘Ribosome’, ‘Oxidative phosphorylation’, and ‘Phagosome’ dominated the top enriched pathways. In addition, a pathway enrichment analysis of either upregulated or downregulated genes from each comparison was conducted (Supplementary File S3). The dominant biological processes in each toxin biosynthesis stage are summarized in Supplementary Figure S9.

### 2.5. Genes Related to Toxin Biosynthesis

Among all the unigenes, a total of 138 homologs of 15 cyanobacterial Sxt proteins were identified: *sxtA*, *sxtB*, *sxtD*, *sxtG*, *sxtH/T/DIOX*, *sxtI*, and *sxtU*, which directly participate in the STX biosynthesis pathway; *sxtF/M*, which is one of the fourteen core toxin genes; and seven other toxin-related genes, *sxtO*, *sxtP*, *sxtW*, *sxtX*, *sxtZ*, *sxtPER*, and *sxtACT* (Table 2 and Supplementary File S4-1). Of these genes, *sxtP*, *sxtPER*, and *sxtACT* were identified for the first time in dinoflagellates. Most of these unigenes altered insignificantly in abundances throughout the four toxin biosynthesis stages, except for Unigene85805 and CL1596.Contig1 (Supplementary File S4-2). Unigene85805 (annotated as *sxtU*, which encodes short-chain alcohol dehydrogenases in cyanobacteria, catalyzing the terminal aldehyde group to an alcohol group) was downregulated at T9. CL1596.Contig1 (annotated as *sxtW* encoding ferredoxins, which provide electrons for hydroxylation reactions catalyzed by *sxtH/T/DIOX* during the toxin biosynthesis pathway) was also downregulated at T9.

## 3. Discussion

### 3.1. Toxin Biosynthesis within the Cell Cycle

The regulation of toxin biosynthesis in *Alexandrium* spp. by the cell cycle is demonstrated in previous studies [20,25]. Our study indicated that the intracellular toxin content of ACHK-T increased gradually in the early G1 phase and rapidly in the late G1 phase; however, an inconspicuous variation of toxin content was observed in the mid G1 phase and S phase (Figure 1B). Two periods of toxin biosynthesis in ACHK-T differed from other *Alexandrium* species, for which strain or species specific differences as well as different culture conditions could be the explanations [19,23,26]. In addition, toxin was produced in both light and dark periods, indicating that there was no direct correlation between light and toxin biosynthesis. This finding was inconsistent with the previous studies that toxin synthesis is induced or regulated by light [17,28]. In general, the correlation between toxin biosynthesis and the cell cycle in *Alexandrium* is complicated, and more effort should be devoted to studying the diverse toxic *Alexandrium* species and strains.

### 3.2. Variations of Toxin Related Genes at Different Toxin Biosynthesis Stages

Based on TBLASTN searching, a total of 138 homologs of 15 cyanobacterial Sxt proteins were identified in this study (Table 2 and Supplementary File S4-1). *sxtA* and *sxtG* are the pivotal genes participating in the first two steps of the toxin biosynthesis pathway, and have been well-studied in dinoflagellates [10,11,15]. As the starting gene of toxin biosynthesis, the long isoform transcribed by *sxtA* is directly involved in toxin biosynthesis, while the short isoform may be concerned in other biological processes [10,14]. *sxtG* encodes amidinotransferases, which transfer an amidino group from arginine to the product of SxtA [5,11]. In our study, both genes were successfully identified in the four transcriptomes, with eleven unigenes assigned to the *sxtA* long isoform, four to the *sxtA* short isoform, and three to the *sxtG* (Supplementary Table S2). However, expressions of these unigenes varied insignificantly at different toxin biosynthesis stages, indicating that these two genes might not be regulated at the transcriptional level during toxin biosynthesis. For other toxin-related genes, only *sxtU* and *sxtW* altered in abundance at some stages (Supplementary File S4-2). These two genes encode short-chain alcohol dehydrogenases and ferredoxins, which catalyze the reactions of the eighth and tenth steps of the toxin biosynthesis pathway [5,6]. Both genes were significantly downregulated at T9, but these decreases might be related to different light conditions, considering a more active toxin biosynthesis at T9 compared to that at T5. Therefore, light produced little effect on toxin production at the transcriptomic level. Further investigation is required to unveil whether this regulation occurs at the translational or post-translational level. In addition, according to the previous studies on phylogenetic analysis of *sxt* genes from dinoflagellates and cyanobacteria, several *sxt* genes are not specific to the toxin biosynthesis pathway, or some have been functionally substituted by other gene products [15,29]. Therefore, the variations of *sxtW* and *sxtU* expressions might be related to other biological processes rather than toxin biosynthesis.

**Figure 4:**
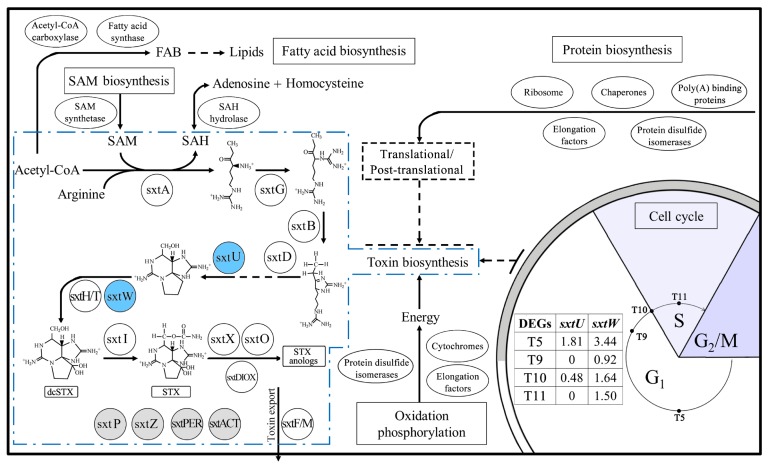
The scheme shows the toxin biosynthesis pathway as well as significantly altered genes and biological processes within a cell cycle. The revised toxin biosynthesis pathway is shown in the blue dotted line box (cited and modified from previous reports [6,9,43]). All of the identified *sxt* genes are labeled with solid circles: the blue circles show the *sxt* genes with significant variations of expression within the cell cycle; the grey circles represent the *sxt* genes without exact functions in toxin biosynthesis of dinoflagellates. The alterations of genes and pathways indicated a translational or post-translational regulation of toxin biosynthesis in *A. catenella*. The abbreviations are: SAM: *S*-adenosyl-methionine; SAH: *S*-adenosyl-homocysteine; FAB: fatty acid biosynthesis.

In cyanobacteria, toxin biosynthesis is hypothesized to be post-translationally regulated based on the expression variations of SxtS and SxtC in a toxin-producing and a nontoxic strain of *Anabaena circinalis* [30,31]. However, only 10–27% of genes are regulated at the transcriptomic level in dinoflagellates [32,33,34], and some genes might be regulated at the post-transcriptional level [35,36]. In addition, some physiological processes such as bioluminescence, carbon fixation, and photosynthesis may be regulated translationally [37,38,39]. In a recent study, both the toxin content and toxin biosynthesis rate in *A. catenella* ACCC01 were found to vary with growth phases, but the expressions of *sxtA4* show inconspicuous variations [40]. A possibility for this discrepancy might be a result of the translational or post-translational regulation of *sxtA4*. Furthermore, the quantities of *sxtA* and *sxtG* mRNA are not correlated with intracellular toxin concentration in *A. minutum* under nitrogen or phosphorus limiting conditions [41]. In our study, the intracellular toxin content at four stages within the cell cycle varied significantly, indicating an active toxin biosynthesis of *A. catenella*, especially during the dark period. In contrast, however, most of the identified toxin-related genes showed insignificant variations among different toxin biosynthesis stages. This result was similar to the previous speculation [42], and, therefore, it is likely that toxin biosynthesis of *A. catenella* might be regulated by translational or post-translational mechanisms (Figure 4). In the future, more efforts should be devoted to differentiating the exact regulation mechanisms of toxin biosynthesis in dinoflagellates.

### 3.3. Other Pathways Potentially Related to Toxin Biosynthesis

Comparing all of the DEGs from the samples of the four stages, 297 genes were identified to be co-expressed, and their expressions undulated among the comparisons (Figure 2A). These genes participated mainly in biological processes such as ribosome functioning, RNA transport, the mRNA surveillance pathway, and protein processing in endoplasmic reticulum. Based on the functional classification, these co-expressed DEGs were assigned to various functions: protein metabolism, chaperone, carbohydrate and energy metabolism, and the oxidation-reduction process (Figure 2C).

Protein synthesis is a complex biological process in organisms, including peptide chain elongation, protein folding, and post-translational modification. Out of the 297 DEGs, 23 were annotated as either the small or the large ribosomal subunits, and six unigenes were assigned to the elongation factors (EFs) that promote and guarantee the accurate elongation of peptide chains during translation in ribosomes [44,45]. Some unigenes were also assigned to protein disulfide isomerases and poly(A) binding proteins, which govern the stability and translation of mRNA [46]. Moreover, the protein folding process requires various chaperones involved in new protein folding, avoiding misfolding, and inhibiting aggregation [47]. In our study, 10 unigenes were identified to encode HSP70, BiP, C-169, chaperonin 10, and calreticulin, and their expressions varied at different toxin biosynthesis stages. The fluctuating expressions of these genes indicated that protein synthesis might be accompanied by toxin biosynthesis within the cell cycle.

Besides those genes related to protein synthesis, genes participating in energy metabolism (carbon fixation and nitrogen metabolism) and carbohydrate metabolism (citrate cycle, glycolysis, and amino acid metabolism) were downregulated significantly at T9. It is reasonable that these basic metabolic activities declined when the cells entered the dark period, which led to a lowered rate of protein synthesis. In the T9-vs-T10 comparison, the expressions of most of the toxin genes varied insignificantly, although toxin was synthesized actively during this period. We proposed that toxin biosynthesis might be regulated by a translational or post-translational mechanism. Accordingly, the enhancement of genes related to protein synthesis was likely to provide enough proteins or enzymes to promote toxin biosynthesis at T10. Subsequently, cells entered the S phase from T10, at which stage nucleotide metabolism became more active while the toxin production was inactive, and protein synthesis was again depressed. Moreover, ribosome biogenesis, which is the process of manufacturing ribosomes, was downregulated significantly at T10, which might account for the depressed activities of protein synthesis at T11.

In addition, three DEGs were annotated as putative cytochrome P450-like protein precursor, F-type H-ATPase beta subunit and cytochrome c oxidase subunit, and these genes are related to oxidation phosphorylation. ATP, as the main energy source for the majority of metabolic pathways, was synthesized through oxidation phosphorylation by utilizing energy released from oxidation reactions in intracellular mitochondria. Several other ATP synthesis related genes were also identified, such as adenylate kinase and adenosine kinase. The fluctuating expressions of these genes indicated a diverse demand of energy for protein synthesis as well as toxin biosynthesis at different stages in the cells.

As one of the precursors involved in the STX pathway, *S*-adenosyl-methionine (SAM) contributes a methyl group to the starting of toxin biosynthesis, and the reaction produces *S*-adenosyl-homocysteine (SAH) which can also be hydrated reversibly from adenosine and homocysteine by *S*-adenosyl-homocysteine hydrolase (SAHH). SAHH functions potentially in regulating the SAM pathway and intracellular SAH concentration, and its encoding gene is thought to be indirectly related to toxin biosynthesis. In our study, several unigenes assigned to SAM synthetase (SAMS) and SAHH were identified, and their expressions altered with the toxin content. The upregulation of the SAMS gene at T10 might provide more SAM for the methyl utilization in toxin biosynthesis, while the enhancement of *Sahh* might assist hydrating the excessive SAH produced by the first step of active toxin synthesis (Figure 4).

Fatty acids and lipids are known to function as constituents of biological membranes or energy storage in cells, and are essential for cell growth [48]. The biosynthesis of free fatty acid can serve as a regulator of the cell cycle in some organisms, for instance, the dinoflagellate *Symbiodinium* [49]. In the heterotrophic dinoflagellate *Crypthecodinium cohnii*, fatty acid biosynthesis (FAB) is coupled to the cell cycle, and sufficient lipids are possibly synthesized before the G2/M phase [50]. Generally, FAB is initiated from acetyl-CoA through the reactions catalyzed by acetyl-CoA carboxylase (ACC) and fatty acid synthase (FAS) [48], while acetyl-CoA was also identified as one of the precursors for STX biosynthesis in an in vitro biosynthesis study [42]. In our study, three unigenes were annotated as ACC, and they were downregulated at T9 compared to T5. Four genes were assigned to FAS, of which two were downregulated at T9 and T10 (one unigene in each), while they were upregulated at T11 compared to T10. In addition to these DEGs, the KEGG pathway enrichment analysis revealed that the FAB process was downregulated at T10 and upregulated at T11, both of which were in the phases before the cells entered the G2/M phase (T12/06:00) (Supplementary File S3). The enhancement of FAB at T11 was consistent with that in *C. cohnii*, indicating an active preparation of lipids for cell division in ACHK-T, while at T10, since acetyl-CoA serves as the precursor in both FAB and toxin biosynthesis, it is therefore conceivable that more acetyl-CoA might flux to rapid toxin production, which will depress FAB (Figure 4).

## 4. Conclusions

This study, for the first time, compared the transcriptome profiles of *A. catenella* (ACHK-T) cells at different toxin biosynthesis stages within the cell cycle. The study identified 138 homologs of 15 toxin genes, but most of them varied insignificantly at different toxin biosynthesis stages, which was inconsistent with the variations of intracellular toxin content, indicating that toxin biosynthesis was not regulated at transcriptional level. Protein synthesis and energy metabolism proceeding simultaneously with toxin biosynthesis indicated an active translation process during toxin production. Taken together, our results suggested that the toxin biosynthesis of *A. catenella* might be regulated translationally or post-translationally. Further investigation is required to determine the toxin genes and their relationship to toxin production in other STX-producing dinoflagellates, and subsequently underlying the exact regulation mechanisms involved in toxin biosynthesis.

## 5. Materials and Methods

### 5.1. Culture Conditioning and Sample Collection

The toxic strain of *A. catenella* (ACHK-T) was provided by the Collection Center of Algae, Xiamen University, China. Cultures were maintained in K medium [51] at 20 °C under a 14:10 h light: dark photoperiod at a photon flux of approximately 100 μmol·m^−2^·s^−1^ provided by fluorescent lamps (Philips, Amsterdam, The Netherlands).

The synchronization of the ACHK-T cells was conducted using the approach previously reported in [52]. The cells were grown in a 5 L flask containing 4 L K medium, and the active cells floating in the upper layer of the flask were transferred into fresh medium every four days for a month. Finally, these synchronized cultures were transferred into three 5 L flasks and grown for two days. The diel sample collection was then carried out every 2 h from the beginning of the light period (08:00) and lasted for 24 h. One milliliter (1 mL) of culture medium was collected and fixed with Lugol’s solution for microscopical cell counting, and 50 mL of culture medium was collected for toxin and FCM analysis, respectively. The medium was centrifuged at 8000× *g* at temperature for 10 min, followed by the removal of the supernatant. The cells pellets for toxin analysis were stored directly at −20 °C, while the pellets for FCM were resuspended in 1 mL 70% ethanol and stored at −20 °C until use.

Our study focused on the toxin biosynthesis within the cell cycle, and thus four samples at different toxin biosynthesis stages were selected for transcriptomic analysis. Based on the preliminary experiments, 500 mL of culture medium from each flask was collected for RNA extraction at 16:00 (T5), 00:00 (T9), 02:00 (T10), and 04:00 (T11) by centrifugation at 8000× *g* at temperature for 10 min. The cell pellets were resuspended in 1 mL TRIzol and stored at −80 °C. T5 was the middle time point where the toxin content increased slowly; T9 was the time point where the toxin content increased rapidly; and T10 and T11 were the transition and termination time points of this rapid increase. The first three time points were in the G1 phase, and T11 was in the S phase.

### 5.2. FCM and Intracellular Toxin Analysis

The FCM samples collected above were centrifuged at 10,000× *g* for 5 min at room temperature, followed by twice washing with phosphate buffered saline (1× PBS, pH = 8.0), and then centrifuged as described above to pelletize the cells. The pellets were subsequently suspended with 100 μL RNase A (100 μg·mL^−1^, Sigma, St. Louis, MO, USA), and incubated at room temperature for 10 min. One milliliter (1 mL) 1 × PBS containing 2.5 mg·mL^−1^ propidium iodide (PI, Sigma, St. Louis, MO, USA) was then added into the resuspended cells, which were then incubated for 1 h at 37 °C. The DNA content analysis of the PI stained cells was conducted on an Epics XL flow cytometer (Beckman Coulter, Miami, FL, USA) with excitation and emission wavelengths at 488 and 635 nm. The relative DNA content was analyzed with MultiCycle software (Version 4.0, Phoenix Flow Systems, San Diego, CA, USA) to obtain the cell cycle profiles for the diel samples.

For toxin extraction, 0.5 mL 50 mM acetic acid was added into the samples to suspend the cell pellets, and the resuspension was then sonicated with short pulses of 3 s for two successive sonication cycles under a power of 15 W (Model 450, Branson Ultrasonics, Danbury, CT, USA). The extracts were collected using centrifugation at 10,000× *g* for 30 min and then filtered through a 0.22 μm MILLEX filter (Millipore, Billerica, MA, USA). The intracellular toxin analysis was carried out using HPLC (HP1100, Agilent, Santa Clara, CA, USA) with post column derivatization using an Inertsil C8-5 column (GL Science, Tokyo, Japan) (15 cm × 4.6 cm) as reported in [53]. The two predominant toxins produced by ACHK-T, C1/C2 and GTX1/4, were examined [14]. Toxin standards for C1/C2 and GTX1/4 were purchased from the National Research Council, Halifax, NS, Canada, and were utilized to quantify the toxin content of each sample. The toxin contents were determined by comparing the peak area for each toxin with that of the standards and were reported as the mean of biological triplicates. The significant differences of the means were tested by one-way analysis of variance (ANOVA) at *p* < 0.05 using SPSS 16.0 (SPSS Inc., Chicago, IL, USA).

### 5.3. RNA Isolation

The RNA samples were homogenized in liquid nitrogen and the total RNA was isolated using TRI-Reagent (MRC, Cincinnati, OH, USA) combined with a QIAGEN RNeasy Mini kit (Qiagen, Valencia, CA, USA) as reported in [54]. To remove potential DNA contaminant, the total RNA was treated with RQ1 RNase-free DNase (Promega, Madison, WI, USA). The RNA concentration was measured using a Qubit^®^ RNA Assay Kit in a Qubit^®^ 2.0 Flurometer (Life Technologies, Carlsbad, CA, USA). The RNA integrity was examined using an RNA Nano 6000 Assay Kit of the 2100 Bioanalyzer RNA Nanochip (Agilent Technologies, Santa Clara, CA, USA). For each sample, aliquots of the total RNA from the triplicates were pooled, and a total of 10 μg RNA was then used to construct the sequencing library [55,56,57,58].

### 5.4. Transcriptome Sequencing

In order to construct libraries, mRNA was enriched with poly-T oligo-attached magnetic beads from the extracted total RNA. Subsequently, mRNA was fragmented into smaller pieces using divalent cations under elevated temperature in an Illumina proprietary fragmentation buffer. First strand cDNA was synthesized with SuperScript II reverse transcriptase and random oligonucleotides using these short fragments as templates. Second strand cDNA was then generated with the GEX second strand buffer, RNase H, and DNA Polymerase I. These double stranded cDNA fragments were further subjected to end reparation, followed by adenylation of the 3′ ends, and ligation with Illumina paired-end adapters. In order to select cDNA fragments of preferentially 200 bp length, adapter ligated fragments were purified and enriched using an Illumina PCR Primer Cocktail in a 10 cycle PCR reaction to create the final libraries. The quality and quantity of the cDNA libraries were measured using the Agilent Bioanalyzer 2100 system (Agilent Technologies, Santa Clara, CA, USA). Finally, the libraries were sequenced on an Illumina Hiseq 2000 platform.

### 5.5. De Novo Assembly and Functional Annotations

All of the the raw reads produced from the Illumina sequencing were first filtered to obtain high quality clean data by removing adapter sequences, reads containing adapters, and low quality reads. The clean reads were deposited in the SRA database of GenBank with the BioProject accession number PRJNA371231. Based on these clean reads, de novo assembly was performed using the Trinity short reads assembling program. The generated unigenes were then further filtered and clustered to remove redundant sequences using the TGICL software [59] with the parameters “−l 40 –c 10 –v 20”. Subsequently, the filtered sequences were translated into amino acids to search by BLASTX against the NR, KEGG, COG, and Swiss-Prot databases, with an e-value threshold of 1 × 10^−5^. The best BLAST result was assigned to the putative functional annotation of each unigene. Based on the NR annotation results, the GO annotation was carried out using Blast2GO software [60], and the GO functional classification was performed on the web-based WEGO program [61] for all of the unigenes.

### 5.6. DEGs Analysis

The uniquely mapped fragment back to a specific unigene was counted by mapping fragments to the assembled unigenes using the Short Oligonucleotide Alignment Program (SOAP) software (SOAP2, version 2.20, BGI, Shenzhen, China) [62]. The calculation of the unigene expression level was then normalized using the fragments per kilobase of transcript per million mapped fragments, to eliminate the influence of variation in gene length and sequencing discrepancies in the gene expression calculation [63]. Using a comparison algorithm developed by the Beijing Genomics Institute (BGI, Shenzhen, China), the unigenes were defined as DEGs with a fold change ≥2 and FDR ≤0.001 [64,65,66].

The GO enrichment analysis of the DEGs was carried out using GO::TermFinder [67]. All of the DEGs were mapped to each term of the GO database and the number of unigenes possessed by each term was calculated, followed by a hypergeometric test to identify significantly enriched GO terms. Based on the Bonferroni Correction, GO terms with a corrected *p*-value (*q*-value) ≤ 0.05 were defined to be significantly enriched. The functional enrichment analysis of KEGG identified significantly enriched pathways in DEGs compared with the whole transcriptome background. After a hypergeometric test and multiple testing correction, all of the metabolic pathways with a *q*-value ≤ 0.05 were reported as significantly altered.

### 5.7. Identification of Sxt Genes

To identify genes potentially related to toxin biosynthesis, TBLASTN was carried out against all of the assembled unigenes using the search strategy based on amino acid sequences as reported in [10,15]. The cyanobacterial Sxt proteins encoded by the *sxt* cluster from *Cylindrospermopsis raciborskii* T3, *Dolichospermum circinale* AWQC131C (formerly *Anabaena circinalis* AWQC131C), *Aphanizomenon* sp. NH-5, *Microseira wollei* (formerly *Lyngbya wollei*), and *Raphidiopsis brookii* D9 were used as queries under a threshold smaller than 1 × 10^−5^ [9].

## Figures and Tables

**Table 1:** Summary statistics of functional annotation.

Database	Number of Unigenes
NR	72,936
NT	8091
KEGG	54,818
Swiss-Prot	49,749
GO	15,432
COG	45,162
At least in one database	74,261

**Table 2:** Blast analysis of potential STX genes in *Alexandrium catenella* (e-value threshold: 1 × 10^−5^). The detailed results are shown in Supplementary File S4-1.

STX Gene	Putative Function	*A. catenella* Unigenes
*sxtA*	Aspartate aminotransferase	17
*sxtB*	Cytidine deaminase	2
*sxtD*	Sterole desaturase	1
*sxtF/M*	Toxic compound efflux protein	2
*sxtG*	Amidinotransferase	3
*sxtH/T/DIOX*	Phenylpropionate dioxygenase	15
*sxtI*	O-carbamoyltransferase	4
*sxtO*	Adenylylsulfate kinase	1
*sxtP*	STX-binding protein	2
*sxtU*	Short-chain alcohol dehydrogenase	66
*sxtW*	Ferredoxin	5
*sxtX*	Cephalosporin hydroxylase	1
*sxtZ*	Two-component sensor histidine kinase	14
*sxtPER*	Permease	1
*sxtACT*	Acyl-CoA dependent acyltransferase	4

## Supplementary Materials

The following are available online at The following are available online at www.mdpi.com/2072-6651/9/7/213/s1, Figure S1: Length distribution of all unigenes, Figure S2: Summary of homology analysis against the NR database, Figure S3: COG classification of *A. catenella* unigenes, Figure S4: Histogram presentation of GO classification for all unigenes, Figure S5: KO annotations of unigenes, Figure S6: KEGG pathway annotation, Figure S7: KEGG classification of unigenes based on the secondary pathway hierarchy, Figure S8: GO classification of DEGs in each comparison, Figure S9: The proposed scheme illustrating cellular processing in different toxin biosynthesis stages within a cell cycle, Table S1: Summary of sequencing output and de novo assembly, Table S2: Identified *sxtA* and *sxtG* and their expressions, File S1: GO enrichment analysis of all DEGs from each comparison, File S2: KEGG enrichment analysis for T5-vs-T9, File S3: KEGG enrichment analysis for up-regulated genes in T5-vs-T9, File S4-1: Searching of *sxt* genes by TBLASN based on sequences from five cyanobacteria species, File S4-2: Expression statistics and sequences of identified *sxt* genes, File S5: Annotations of all assembled genes, File S6: The one-way ANOVA analysis results of toxin content.
