# Supplementary material for: Whole Transcriptomic Analysis Provides Insights into Molecular Mechanisms for Toxin Biosynthesis in a Toxic Dinoflagellate Alexandrium catenella (ACHK-T)
Source: Toxins (Basel). 2017 Jul 5;9(7):213. doi: 10.3390/toxins9070213 (PMC5535160; doi:10.3390/toxins9070213)
Supplement: Supplementary file 1 [file toxins-09-00213-s001.zip › toxins-203236-supplementary/toxins-203236 supplementray.pdf]

Supplementary Materials: Whole Transcriptomic Analysis Provides Insights into Molecular Mechanisms for Toxin Biosynthesis in the Toxic Dinoflagellate *Alexandrium catenella* (ACHK-T)

Yong Zhang, Shu-Fei Zhang, Lin Lin and Da-Zhi Wang

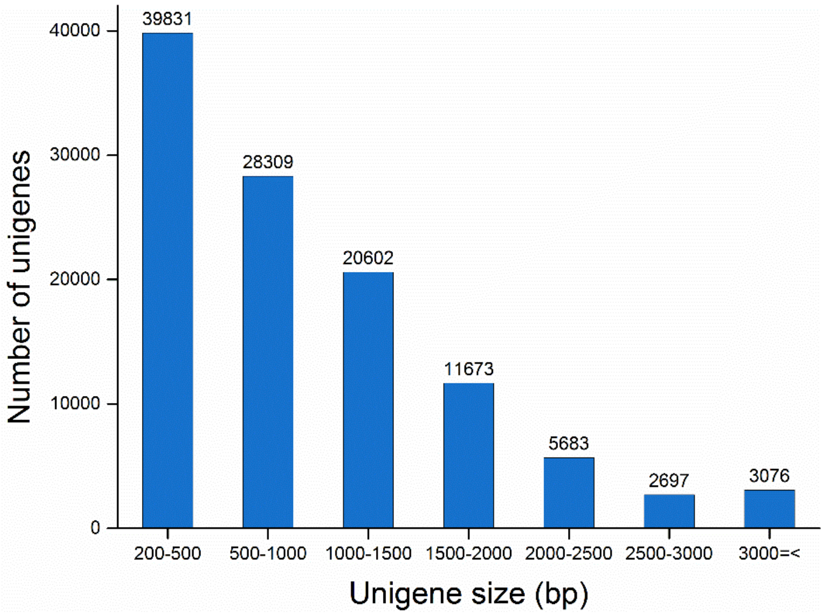

Figure S1. Length distribution of all unigenes. The Y-axis indicated the number of unigenes in the corresponding size of sequence length.

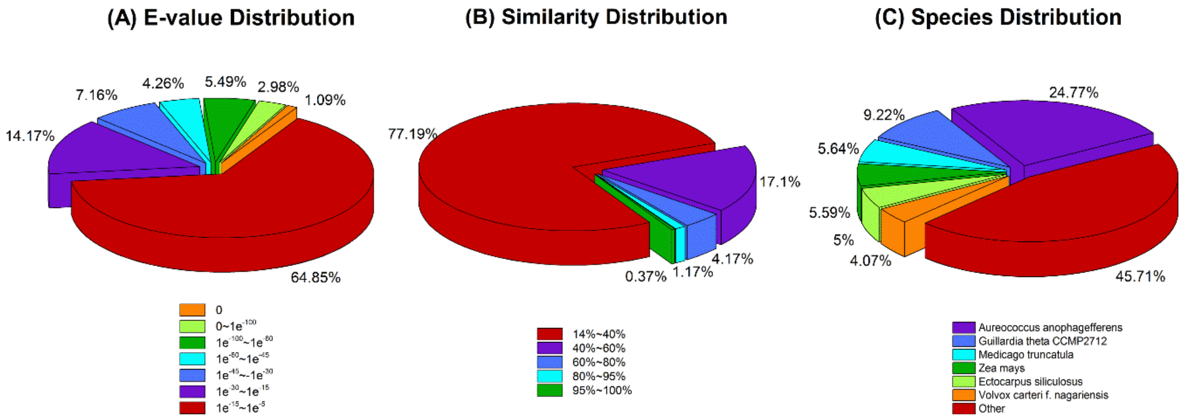

Figure S2. Summary of homology analysis against the NR database. All the unigenes with annotations under a threshold of e-value ≤ 1 × 10^{-5} were analysed for **(A)** e-value distribution of BLAST hits. **(B)** Similarity distribution of the top BLAST hits. **(C)** Species distribution.

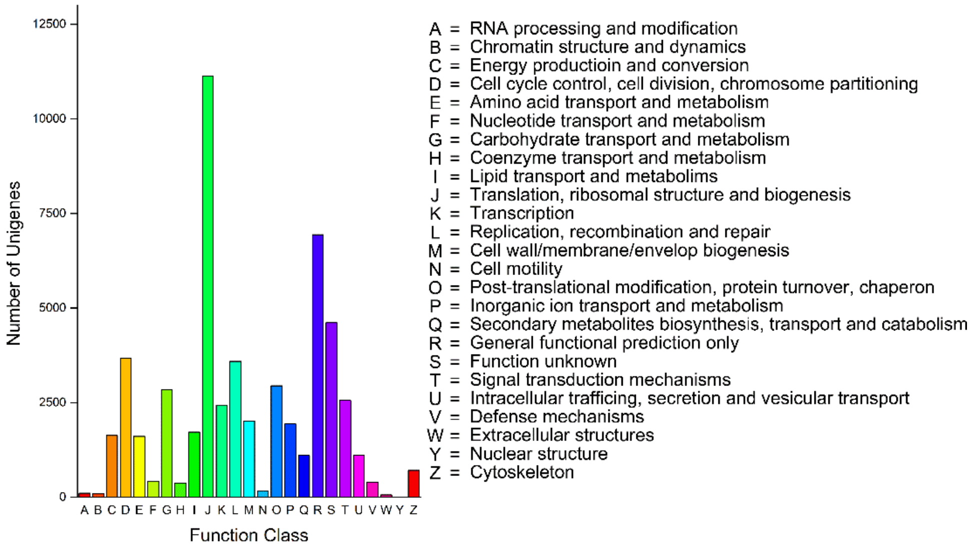

Figure S3. COG classification of *A. catenella* unigenes. Totally, 45,162 unigenes were categorized into 25 COG groups.

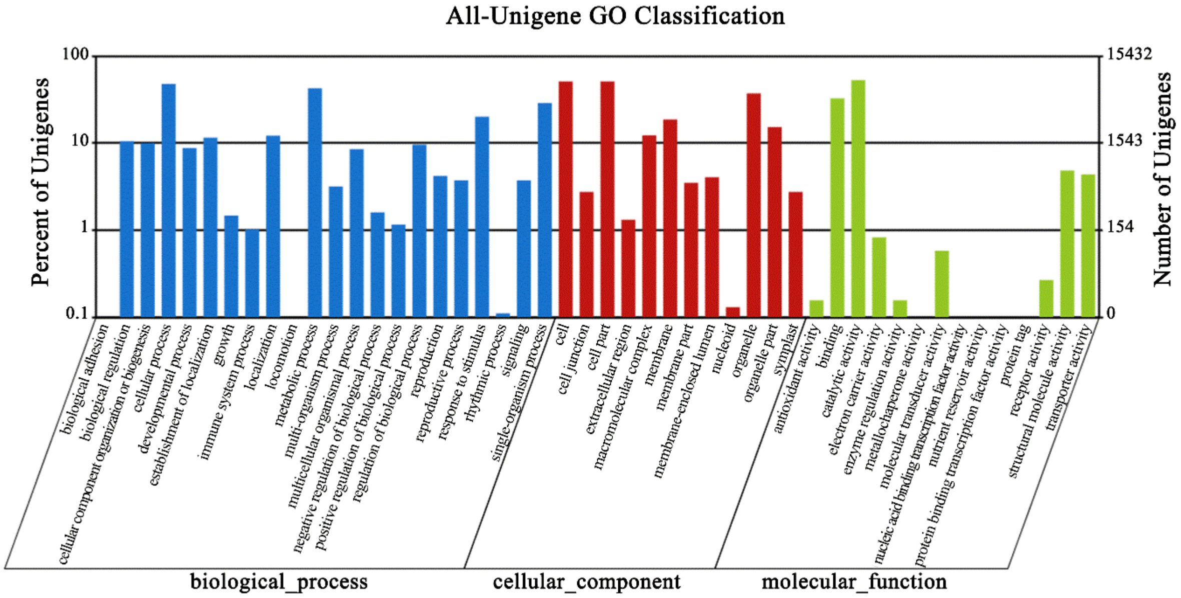

Figure S4. Histogram presentation of GO classification for all unigenes. A total of 15,432 unigenes were assigned to three main categories: biological process, cellular component and molecular function. The Y-axis indicated the percent and number of unigenes within each subcategories.

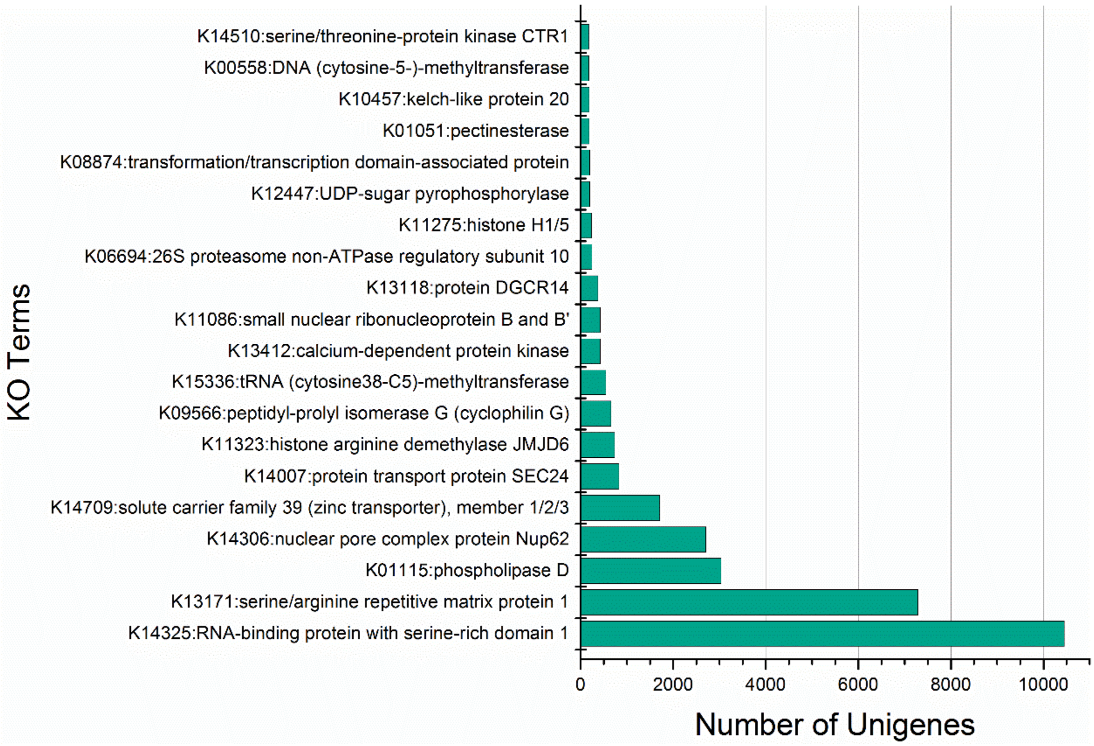

Figure S5. KO annotations of unigenes. Top 20 KO terms with number of unigenes assigned to each term.

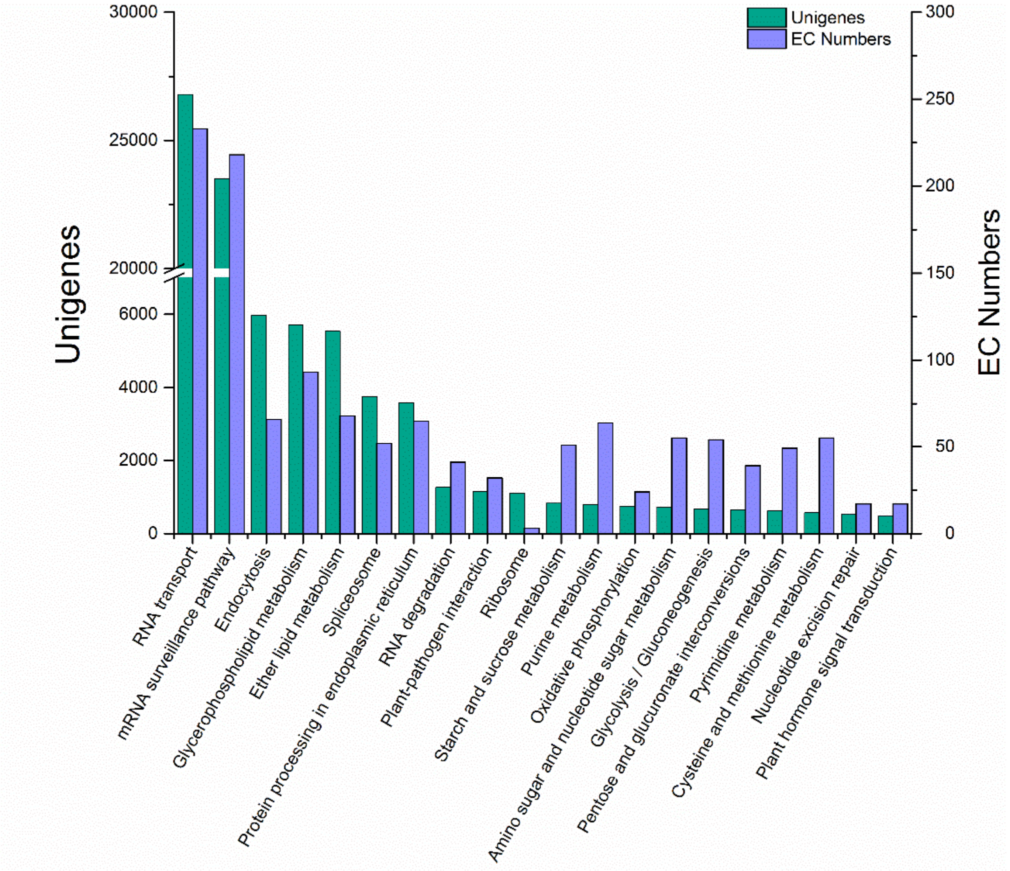

Figure S6. KEGG pathway annotation. The top 20 represented KEGG pathways with numbers of unigenes and EC number within each pathway.

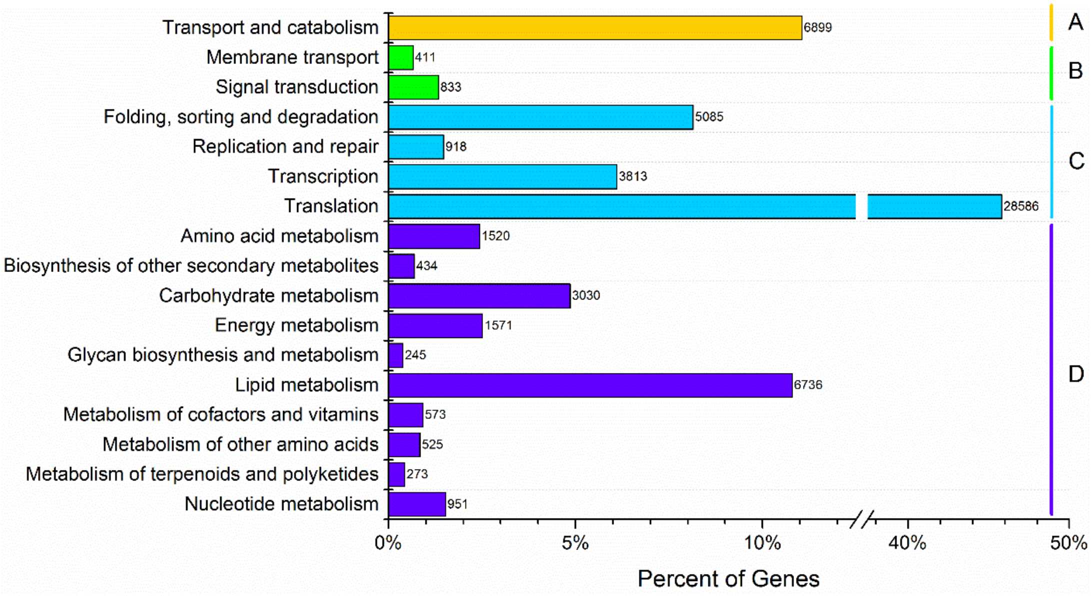

Figure S7. KEGG classification of unigenes based on the secondary pathway hierarchy. Letters correspond to the primary hierarchy, A: Cellular Processes; B: Environmental Information Processing; C: Genetic information Processing; D: Metabolism.

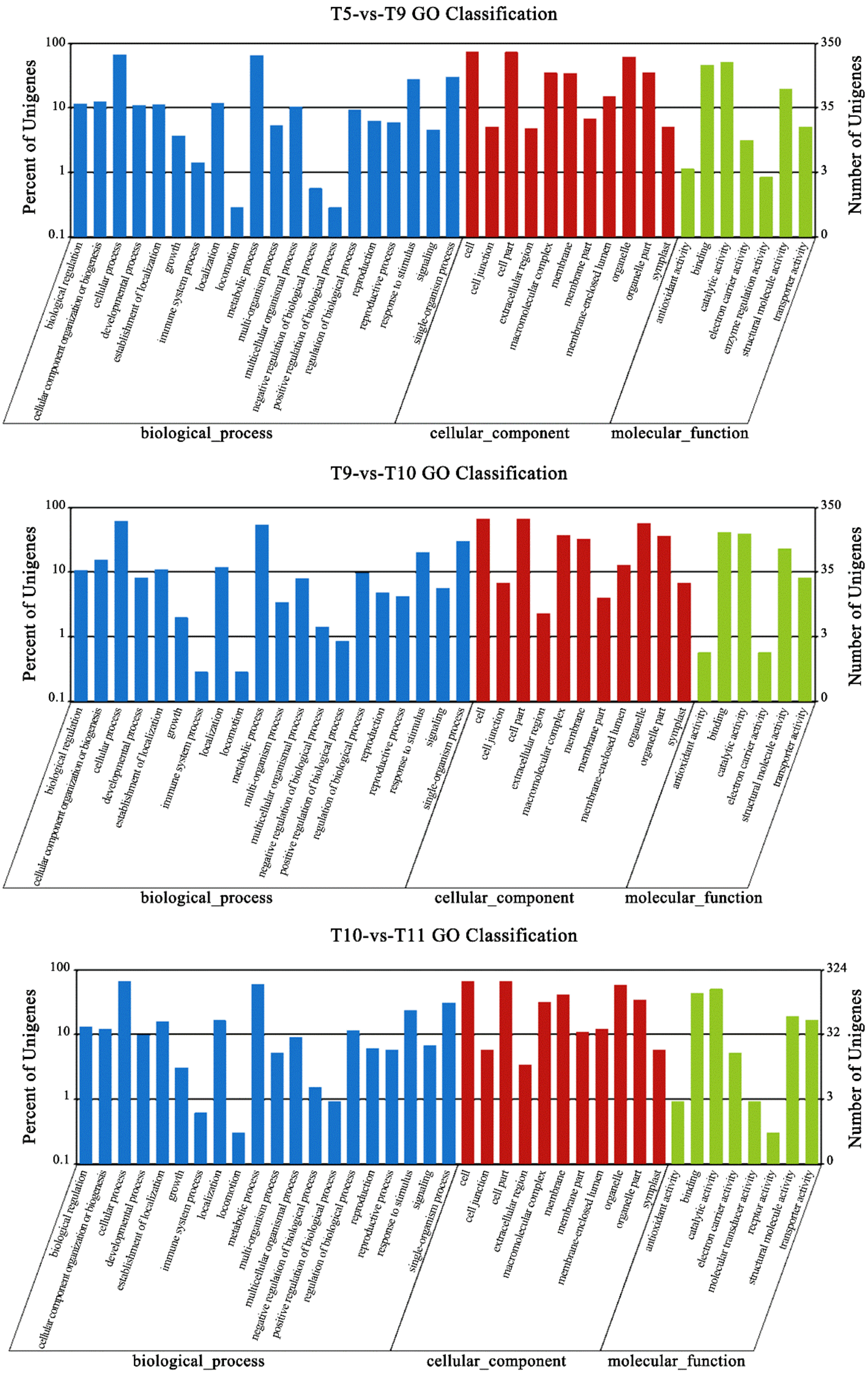

Figure S8. GO classification of DEGs in each comparison. For T5-vs-T9, T9-vs-T10 and T10-vs-T11831, 776 and 760 DEGs were categorized, respectively.

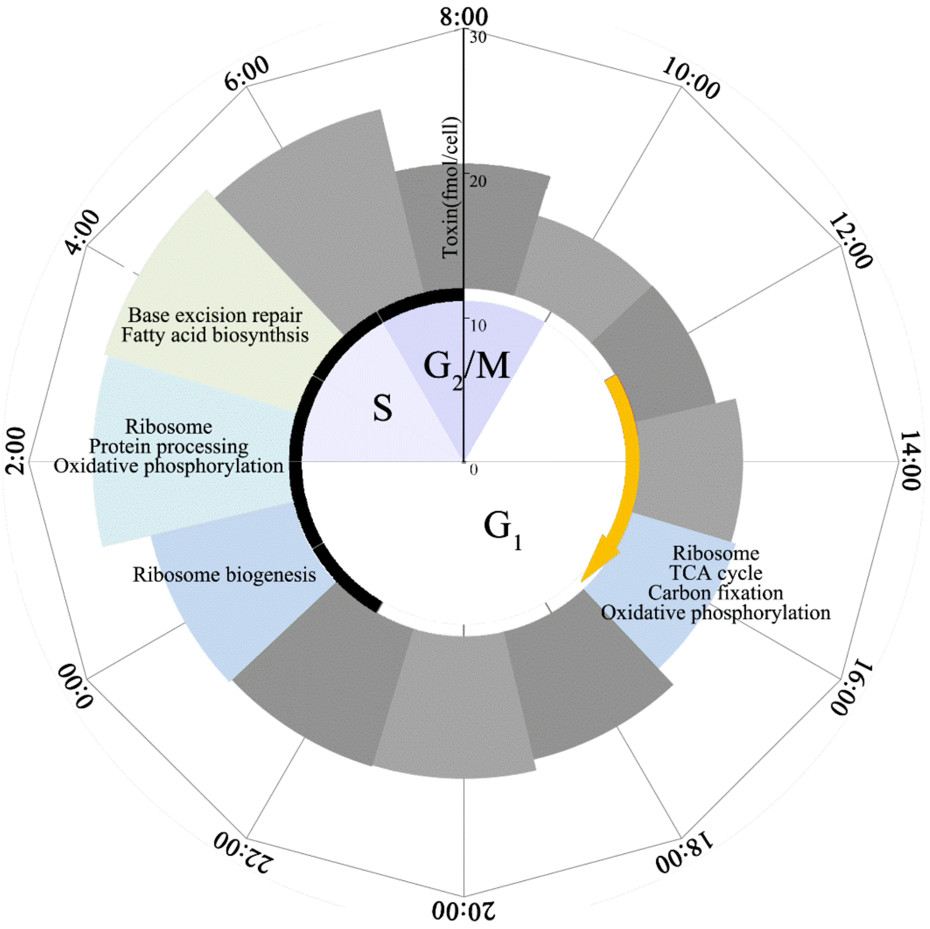

Figure S9. The proposed scheme illustrating cellular processing in different toxin biosynthesis stages within a cell cycle. The area of grey and light-coloured sectors represented the single cellular toxin concentration in each sampling time point.

Table S1. Summary of sequencing output and de novo assembly.

Items	Value
Total number of raw reads	480,981,760
Total number of clean reads	435,543,566
Total clean nucleotides (Gb)	43.55
Q20 percentage (%)	96.50~96.69
GC percentage (%)	60.75~60.93
Total number of transcripts	701,597
Mean length of transcripts	554
N50 length of transcripts	1,106
Total number of unigenes	111,871
Mean length of unigenes	993
N50 length of unigenes	1,442

Table S2. Identified *sxtA* and *sxtG* and their expressions.

Gene_ID	Gene Length	T5_FPKM	T9_FPKM	T10_FPKM	T11_FPKM
sxtA long:					
CL2951.Contig1_All	1537	17.6161	19.0586	18.791	23.0346
CL2951.Contig2_All	1647	22.0835	20.5573	20.1706	20.728
CL2951.Contig3_All	220	21.9956	18.8725	18.5755	19.9163

CL4218.Contig2_All	2037	15.9439	19.966	15.0915	23.0744
CL4218.Contig3_All	1698	5.0104	5.1876	4.6106	5.8232
Unigene14356_All	1749	61.0998	61.0656	55.5126	67.1983
Unigene26180_All	1285	2.6273	2.6777	2.4298	3.5374
Unigene51526_All	1072	29.3726	27.7108	26.9634	29.5729
Unigene68071_All	4200	3.8959	2.7472	3.4274	3.7043
Unigene77178_All	5379	5.1048	4.7891	3.3804	4.1948
Unigene84411_All	908	2.1813	2.1726	2.1745	2.5289
sxtA short:					
Unigene44192_All	1546	3.7706	3.5314	3.3413	4.0012
Unigene51437_All	3095	4.596	4.4988	3.9983	4.9663
Unigene5533_All	3086	8.7884	9.2544	7.3503	10.159
Unigene87971_All	200	1.2379	0.5735	1.6071	0.2343
sxtG:					
CL1611.Contig1_All	1237	4.8762	4.7473	4.6585	4.6597
CL1611.Contig2_All	1254	4.146	4.0061	3.863	3.4941
Unigene10989_All	548	24.1089	37.799	22.6233	17.4877
